# Machine Learning-Based Automated Detection and Quantification of Geographic Atrophy and Hypertransmission Defects Using Spectral Domain Optical Coherence Tomography

**DOI:** 10.3390/jpm13010037

**Published:** 2022-12-24

**Authors:** Gagan Kalra, Hasan Cetin, Jon Whitney, Sari Yordi, Yavuz Cakir, Conor McConville, Victoria Whitmore, Michelle Bonnay, Leina Lunasco, Antoine Sassine, Kevin Borisiak, Daniel Cohen, Jamie Reese, Sunil K. Srivastava, Justis. P. Ehlers

**Affiliations:** Tony and Leona Campane Center for Excellence in Image-Guided Surgery and Advanced Imaging Research, Cole Eye Institute, Cleveland Clinic, Cleveland, OH 44195, USA

**Keywords:** age-related macular degeneration, geographic atrophy, automated segmentation, machine learning, quantitative optical coherence tomography, retinal imaging automation, precision medicine

## Abstract

The current study describes the development and assessment of innovative, machine learning (ML)-based approaches for automated detection and pixel-accurate measurements of regions with geographic atrophy (GA) in late-stage age-related macular degeneration (AMD) using optical coherence tomography systems. 900 OCT volumes, 100266 B-scans, and *en face* OCT images from 341 non-exudative AMD patients with or without GA were included in this study from both Cirrus (Zeiss) and Spectralis (Heidelberg) OCT systems. B-scan and *en face* level ground truth GA masks were created on OCT B-scan where the segmented ellipsoid zone (EZ) line, retinal pigment epithelium (RPE) line, and bruchs membrane (BM) line overlapped. Two deep learning-based approaches, B-scan level and *en face* level, were trained. The OCT B-scan model had detection accuracy of 91% and GA area measurement accuracy of 94%. The *en face* OCT model had detection accuracy of 82% and GA area measurement accuracy of 96% with primary target of hypertransmission on *en face* OCT. Accuracy was good for both devices tested (92–97%). Automated lesion size stratification for CAM cRORA definition of 250um minimum lesion size was feasible. High-performance models for automatic detection and segmentation of GA area were achieved using OCT systems and deep learning. The automatic measurements showed high correlation with the ground truth. The *en face* model excelled at identification of hypertransmission defects. The models performance generalized well across device types tested. Future development will include integration of both models to enhance feature detection across GA lesions as well as isolating hypertransmission defects without GA for pre-GA biomarker extraction.

## 1. Introduction

Geographic atrophy (GA) is a late-stage finding in age-related macular degeneration (AMD) resulting from atrophic changes in the retinal outer layers and retinal pigment epithelium (RPE). Progression to GA, particularly subfoveal GA, results in permanent vision loss, affecting about 8 million people aged 55 years or older in the United States [1]. GA has an exponential increase in prevalence with age and is more prevalent in those of European heritage [2]. Due to the aging population, the number of individuals affected by GA is likely to grow further in the near future [3].

The irreversible nature of the disease makes monitoring an essential part of clinical care of these patients. The Classification of Atrophy Meetings (CAM) consensus, which comprises specialists in age-related macular degeneration, imaging, and retinal history, agreed in 2018 that optical coherence tomography (OCT) is the best tool for identifying and monitoring GA. Spectral domain (SD)-OCT, being a three-dimensional imaging technique, offers several benefits over two-dimensional approaches like color fundus photography and fundus autofluorescence, including thorough characterization of the inner and outer retinal layers at high resolution [4]. The use of an *en face* image complements the standard cross-sectional OCT B-scan allowing for a complete macular review at various depth levels, which may give further anatomic insight into this condition [5]. The CAM consensus guidelines defined complete RPE and Outer Retinal Atrophy (cRORA) based on the presence of following findings on OCT: (i) RPE loss of at least 250 um in greatest diameter, (ii) overlying photoreceptor loss, and (iii) hypertransmission of at least 250 um in greatest diameter. GA lesions not meeting the size criteria are classified as incomplete RPE and Outer Retinal Atrophy (iRORA). 

Currently, no FDA-approved treatment has been demonstrated to effectively prevent, slow, or stop the progression of GA. Yet, clinical trials for a number of promising treatments are now ongoing. The current widely-used standard for GA measurement is fundus autofluorescence (FAF). However, FAF does not provide cross-sectional information related to anatomic structure and perhaps more importantly is not widely available across practices. In current clinical trials, reading centers perform manual segmentation of anatomical boundaries seen in imaging modalities in order to diagnose and quantify GA [6]. This is a time and labor-intensive process, which can are also subject to human bias and potentially error, particularly when dealing with large datasets [6]. 

As new therapies emerge and the paradigm shifts for referral of these patients and ongoing management of these patients, accurate and reproducible automated detection and quantification of GA would facilitate clinician identification of patients who might benefit from therapy and to readily assess disease progression. Machine learning (ML)-based automated segmentation of features has enabled next-generation capabilities for multiple ophthalmic conditions. Thus, an automated method for identifying, segmenting, and quantifying GA would be extremely beneficial for monitoring patients in clinical practice and quantifying the effectiveness of novel treatments in clinical studies [1]. Additionally, these automated approaches have the capacity to swiftly and reliably extract measurable structural properties of GA that can aid in clinical decision making. The current study explores innovative, fully automated approaches to detect the presence of GA and obtain pixel-accurate segmentation of GA lesions using ML-based methods on both the B-scan image and *en face* OCT image.

## 2. Materials and Methods

This analysis was an IRB approved analysis adhered to the tenets of the Declaration of Helsinki and complied with Health Insurance Portability and Accountability Act regulations (HIPAA). All patient health information was stripped from the collected imaging data included in this study in compliance with HIPAA guidelines. 

### 2.1. Imaging and Data Collection

100,266 SD-OCT B-scans and 900 *en face* SD-OCT images captured using Heidelberg Spectralis HRA+OCT (Heidelberg Engineering, Heidelberg, Germany) or Cirrus HD-OCT (Zeiss, Oberkochen, Germany) devices were collected from 900 visits of 341 de-identified subjects with non-exudative AMD with or without GA through the Tony and Leona Campane Center for Excellence in Image-Guided Surgery and Advanced Imaging Research at the Cole Eye Institute of the Cleveland Clinic. Images captured from the Heidelberg device utilized the 97 B-scan macular cube for image capture. The Zeiss device utilized the 512 × 128 imaging protocol for 6 mm × 6 mm macular cube for image capture.

### 2.2. Retinal Layers Ground Truth

OCTs were automatically segmented using a previously validated ML-enabled multi-layer segmentation platform for the following layers: ellipsoid zone (EZ), bruchs membrane (BM), and the retinal pigment epithelium (RPE) for 100,266 SD-OCT B-scans [7,8,9]. Briefly, EZ was measured between the EZ segmentation line and RPE segmentation line. Similarly, RPE was measured between RPE segmentation line and BM segmentation line. In areas of atrophy or absence of these layers, the measured EZ-RPE and RPE-BM thickness were zero as denoted by overlap/complete confluence of EZ, RPE, and BM segmentation lines. These segmentation masks were manually corrected with previously validated two-tier sequential expert image analyst review [7,8,9] such that machine learning based segmentation was first corrected for errors by an expert reader followed by an independent review by a senior expert reader to ensure consistency and quality. Any discrepancies arising in the first two layers of review were reconciled with an analysis director.

### 2.3. GA Ground Truth Mask Generation for B-Scan Model

**Figure 1:**
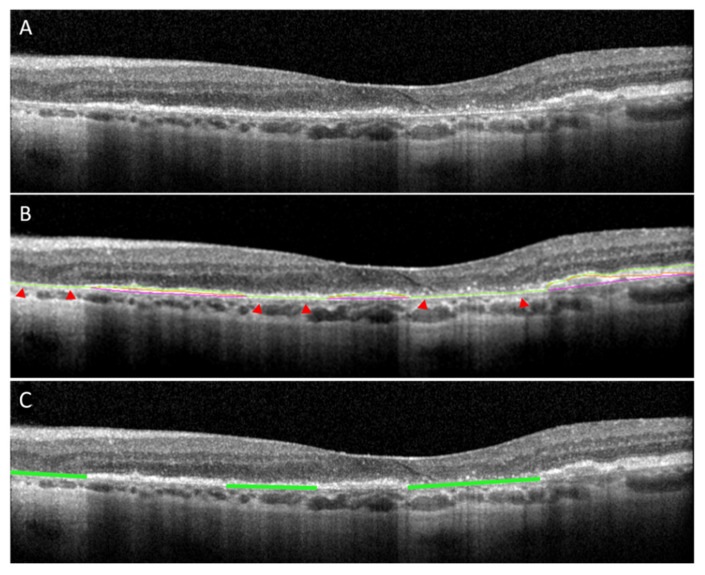
(**A**) Raw B-scan showing areas advanced atrophic changes; (**B**) Multi-layer segmented SD-OCT B-scan from a patient with GA with EZ denoted in green, RPE denoted in orange and BM denoted in pink. Red arrow heads indicate areas of concurrence of the three lines—definition of GA by initial read (i.e., absence of EZ and RPE); (**C**) Final GA ground truth mask overlay for the same B-scan indicated in green. SD-OCT: Spectral Domain Optical Coherence Tomography; GA: Geographic Atrophy; EZ: Ellipsoid Zone; RPE: Retinal Pigment Epithelium; BM: Bruchs Membrane. Image shown was Captured using Spectralis HRA+OCT (Heidelberg Engineering, Heidelberg, Germany) with the 97 B-scan macular cube protocol.

Binary retinal layer segmentation masks were exported for each B-Scan, and complete attenuation of EZ and RPE (i.e., no visible EZ and RPE line, definition of GA by initial read) were identified as areas where the EZ, RPE, and BM segmentation lines were concurrent. This allowed for layer-based segmentation defined regions to generate GA ground truth masks for B-scan model training. Areas where all 3 bands were concurrent were assembled into ground truth masks for GA training. These lines were dilated to a thickness of 10 pixels centered on the BM segmentation line. Figure 1A shows an example of a raw B-scan with findings of GA defined complete loss of the ellipsoid zone, complete loss of retinal pigment epithelium, and presence of hypertransmission into the choroid, as per CAM definitions [4]. Figure 1B shows the complete overlap EZ, RPE, and BM segmentation lines in areas of GA indicated by red arrows. Figure 1C shows the same OCT B-scan with the final GA ground truth mask overlay indicated in green.

### 2.4. GA Ground Truth Mask Generation for En Face Model

**Figure 2:**
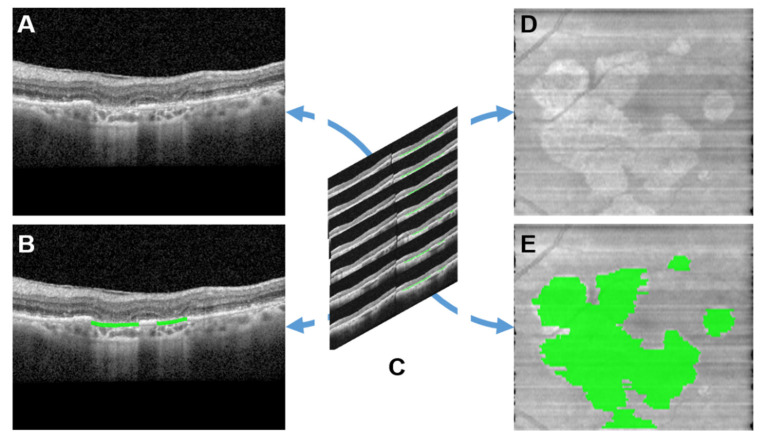
GA ground truth masks as created for (**A**,**B**) Individual B-Scans by obtaining EZ-RPE-Bruchs membrane overlap; (**C**) *En face* images compiled using individual B-Scans; (**D**,**E**) GA *en face* ground truth masks generated for model training. GA: Geographic Atrophy; EZ: Ellipsoid Zone; RPE: Retinal Pigment Epithelium. Raw images shown were captured using Spectralis HRA+OCT (Heidelberg Engineering, Heidelberg, Germany) with the 97 B-scan macular cube protocol.

Original *en face* OCT images were obtained by computing the *en face* projection of the entire OCT macular cube without using any custom segmentation slabs. Ground truth masks for each B-scan were assembled into *en face* ground truth masks using the following method. First, the horizontal dimensions of each scan were sized to fit the width of the original *en face* scans. Each B-Scan corresponded with a single vertical pixel on the y-axis of the *en face* scan, so there was no vertical rescaling necessary. The maximum value for the mask at each x coordinate was mapped to a corresponding ground truth *en face* mask, and the y coordinate was identified by the B-scan slice number, generating a corresponding *en face* ground truth GA map for *en face* model training. The process of generation of GA ground truth masks for *en face* model is summarized in Figure 2. The final GA ground truth mask overlay for *en face* OCT is illustrated in Figure 2E.

### 2.5. Training Data

The training data was comprised of 80% total patients included in the study. Timepoints from patients with multiple timepoints were censored from being in both training and testing datasets. This ensured that all time points of each patient were clustered together in either training or testing datasets but not both allowing a distinct separation between datasets. This data comprised images from both device manufacturers allowing for an opportunity for testing the feasibility of device-agnostic segmentation models. The B-Scan GA ground truth masks and corresponding SD-OCT B-scans were used as training inputs for the B-Scan deep learning GA detection model. The *en face* GA ground truth masks and the corresponding SD-OCT *en face* images were used as training inputs for *en face* deep learning GA detection model. 

### 2.6. Validation Data

The validation data comprised of 10% total patients included in the study. This dataset was used to iteratively assess and improve performance during different epochs of model training.

### 2.7. Testing Data

A holdout test set was curated using 10% of the patients in the dataset. Caution was exercised to ensure that any of the timepoints of the patients in the holdout test set were not used for model training. This previously unseen or hold-out dataset was used to assess the fully trained model to generate performance metrics.

### 2.8. Deep Learning Architecture

The B-Scan and *en face* models were trained using a UNet architecture using images resized to 256 × 256 pixel inputs, kernel width: 5, early training stopping after 7 epochs without validation improvement, a batch size of 40, and 200 samples per epoch. The UNet model itself has approximately 20 million parameters, and 41 layers. The models used a binary cross-entropy loss function, and Root Mean Squared optimizer with a learning rate of 1 × 10^−4^.

### 2.9. Automatic Retraining

After the initial round of training, a sample of 100 patches were selected and profiled for their F-scores, such that the 30th percentile of F scores was identified. Then, the training set was run through the model, and patches with F-scores lower than the 30th percentile were duplicated within the training set, prior to a second round of training using the same model and parameters. This allowed for the model to be fine-tuned to focus on training examples with the worst performance.

### 2.10. Binary Detection of Presence of GA 

Binary detection of presence of GA was tested by assessing if the model prediction had any number of pixels that were labelled as GA in comparison to ground truth. This allows for a global assessment of presence or absence of GA in each B-scan using the B-scan model and the whole OCT volume using the *en face* model.

### 2.11. GA Lesion Size Classifier

**Figure 3:**
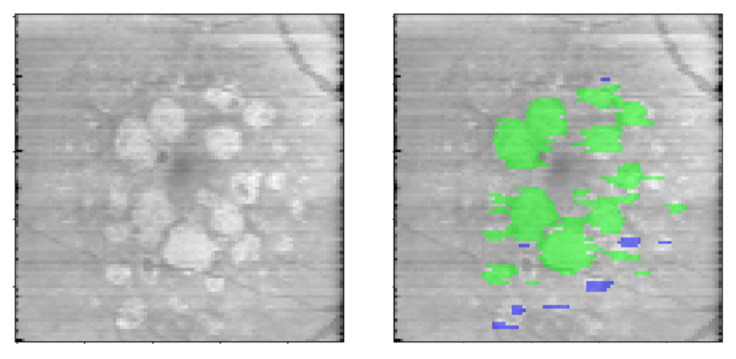
Raw *en face* OCT image (Left); *En face* OCT image with cRORA ground truth mask overlay (shown in green) and GA lesions that did not meet the 250-micron size threshold (shown in blue). OCT: optical coherence tomography; cRORA: complete RPE and outer retinal atrophy; RPE: retinal pigment epithelium. Images were captured using Heidelberg Spectralis HRA+OCT (Heidelberg Engineering, Heidelberg, Germany) with the 97 B-scan macular cube protocol.

As a part of an additional feasibility analysis, GA lesions wider than 250 microns in the greatest diameter on the *en face* GA map were classified as cRORA, per the 250 microns threshold given by the CAM consensus [10]. This classification included non-horizontal linear dimensions for each GA lesion on the *en face* map. Each GA area panning multiple B-scans that was found to be a single continuous lesion on the *en face* map was treated as one lesion for this assessment. The greatest non-linear dimension was obtained for each lesion automatically by utilizing the Skimage.regionprops library in Python and measuring the major axis. The GA lesion masks that met the size threshold of 250 microns were considered to be cRORA (Figure 3). Lesions smaller than 250 microns were considered iRORA.

### 2.12. Statistical Analysis 

The entire test set was evaluated for F-score, accuracy, precision, recall, and True/False Positive/Negative percentile rates for detection of presence of GA and pixel-wise GA area measurement. Pearson’s correlation coefficient (R) and inter class correlation (ICC) coefficient was calculated to compare the ML model outputs and ground truth measurements of GA area.

## 3. Results

### 3.1. The B-Scan Cross-Sectional GA Detector and Quantifier

**Figure 4:**
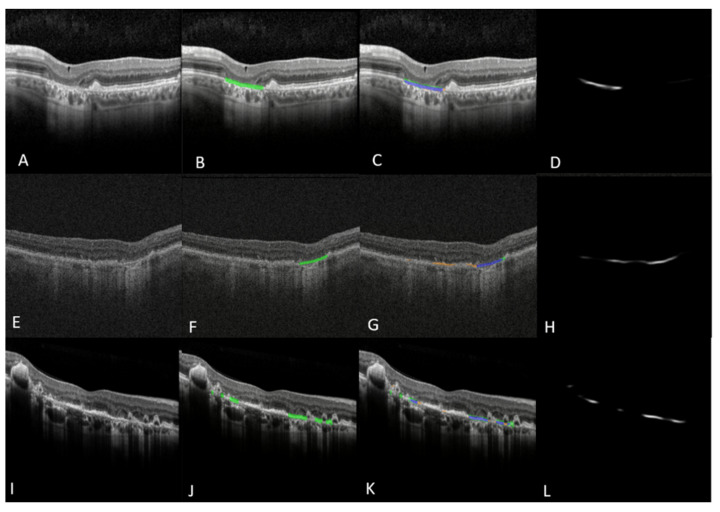
Left panel (**A**–**D**) shows B-Scan model GA segmentation performance as visualized on individual B-Scans of an OCT volume. (**A**) Raw B-scan from a patient with GA; (**B**) Ground truth mask (indicated in green) generated using multi-layer segmentation and concurrence of EZ, RPE, and BM lines; (**C**) B-scan model output overlay with blue areas indicating correct segmentation of GA area, orange areas indicating false positive segmentation and green areas indicating false negative segmentation; (**D**) Gray-scale output of the B-scan model indicating model confidence. Middle panel (**E**–**H**) shows an example of false positive segmentation. Right panel (**I**–**L**) shows an example of false negative segmentation. *GA: Geographic Atrophy; EZ: Ellipsoid Zone; RPE: Retinal Pigment Epithelium; BM: Bruchs Membrane.* Images A–C and I–K were captured using Spectralis HRA+OCT (Heidelberg Engineering, Heidelberg, Germany) with the 97 B-scan macular cube protocol. Images E-G were captured using or Cirrus HD-OCT (Zeiss, Oberkochen, Germany) with the 512 × 128 imaging protocol for 6 mm × 6 mm macular cubes.

The fully automated B-Scan ML model (Figure 1) for detection of GA in an OCT B-scan yielded an f-score of 0.87, accuracy of 91% with a sensitivity of 86% and specificity of 94% (Table 1). The B-Scan ML model for localization and segmentation of GA yielded a pixel-wise accuracy of 0.94 with an f-score of 0.71, sensitivity of 90% and specificity of 90% (Table 2). The model output can be visualized in Figure 4. The prediction of pixel wise area measurements of GA using the B-Scan showed good correlation with the ground truth segmentation of GA area (R = 0.77, *p* < 0.001). ICC of 0.88 (95% CI: 0.82–0.92) was achieved between the predictive and ground truth measurements of GA area. Although the model performance for Cirrus scans was quite good (i.e., accuracy = 92%, sensitivity = 88%, specificity =87%), it did appear to be generally lower than for Spectralis scans (i.e., accuracy = 97%, sensitivity = 96%, specificity = 94%) (Table 3). 

### 3.2. The En Face OCT-Based GA and Hypertransmission Defect Detector and Quantifier 

**Figure 5:**
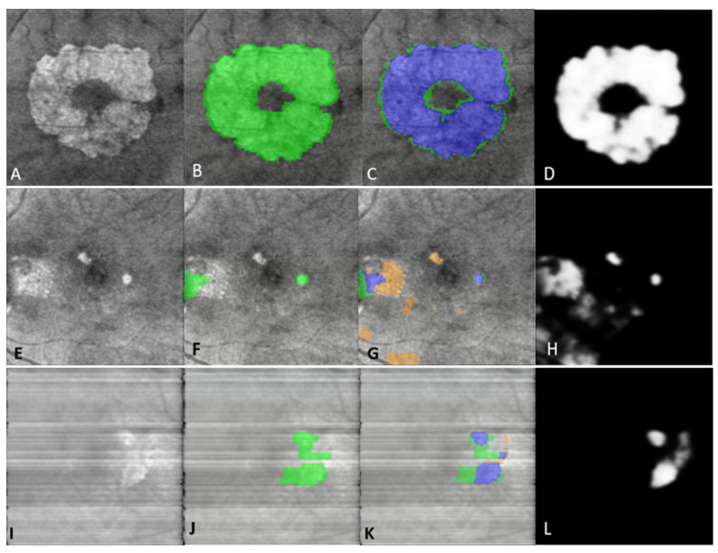
Left panel (**A**–**D**) shows *en face* model GA segmentation performance as visualized on *en face* OCT images. (**A**) Raw *en face* OCT image from a patient with GA; (**B**) Ground truth mask (indicated in green) generated using multi-layer segmentation and concurrence of EZ, RPE, and BM lines, and subsequent compilation of B-Scan masks for the whole volume; (**C**) *en face* model output overlay with blue areas indicating correct segmentation of GA area, orange areas indicating false positive segmentation for GA (but true positive of hypertransmission defects) and green areas indicating false negative segmentation; (**D**) Gray-scale output of the *en face* model indicating model confidence. Middle panel (**E**–**H**) shows an example of false positive segmentation. Right panel (**I**–**L**) shows an example of false negative segmentation. GA: Geographic Atrophy; EZ: Ellipsoid Zone; RPE: Retinal Pigment Epithelium; BM: Bruchs Membrane. Images A-C and E-G were captured using or Cirrus HD-OCT (Zeiss, Oberkochen, Germany) with the 512 × 128 imaging protocol for 6 × 6 mm macular cubes. Images I-K were Captured using Spectralis HRA + OCT (Heidelberg Engineering, Heidelberg, Germany) with the 97 B-scan macular cube protocol.

**Figure 6:**
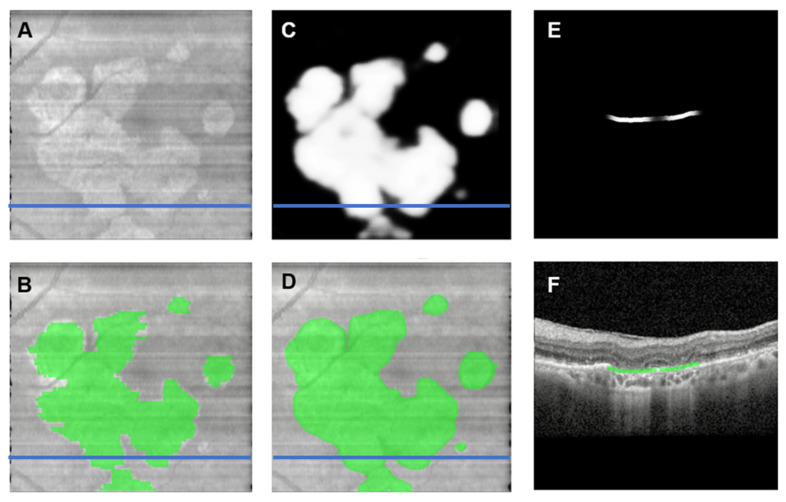
(**A**,**B**) Model training input images; (**C**) Grayscale output from the *en face* ML-model; (**D**) Binary output mask from the *en face* ML-model demonstrating excellenct concordance to the ground truth masks (**B**) with some additional identified areas of hypertransmission defects; (**E**,**F**) Projection of the *en face* model prediction onto the OCT B-scan BM line for GA area prediction. The blue line indicates the position of the B-scan. ML: Machine learning; OCT: Optical Coherence Tomography. Captured using Spectralis HRA+OCT (Heidelberg Engineering, Heidelberg, Germany) with the 97 B-scan macular cube protocol.

**Figure 7:**
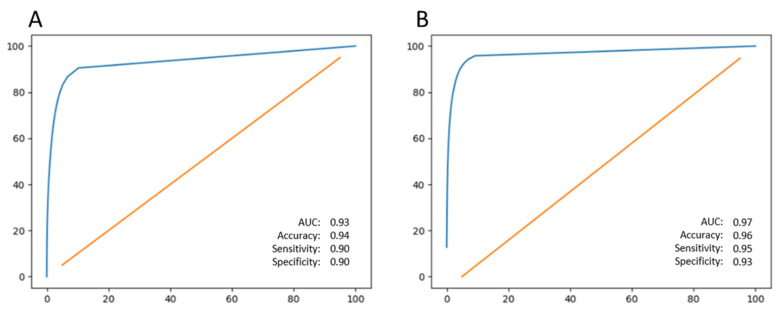
(**A**) Receiver operator curve for automatic pixel-wise GA area segmentation for B-scan model; (**B**) Receiver operator curve for automatic pixel-wise GA area segmentation for *en face* model. The line in orange denotes random chance and the line in blue denotes the model output. GA: Geographic Atrophy.

The fully automated *en face*-based ML model (Figure 2) for detection of GA in an OCT volume yielded an f-score of 0.88, accuracy of 82% with a sensitivity of 97% and specificity of 42% (Table 1). The *en face*-based ML model for localization and segmentation of GA area in an OCT volume yielded a pixel-wise accuracy of 96% with an f-score of 0.82, sensitivity of 95% and specificity of 93% (Table 2). The model output can be visualized in Figure 5. The prediction of pixel wise area measurements of GA using the *en face* model strongly correlated with the ground truth segmentation of GA area (R = 0.93, *p* < 0.001). ICC of 0.95 (95% CI: 0.83–0.92) was achieved between the predictive and ground truth measurements of GA area. The *en face* identified lesions corresponded to areas of hypertransmission defects on the *en face* image. The identified regions were then be projected on individual B-scans using the BM line mask to obtain B-scan level GA area segmentation as shown in Figure 6. The pixel-wise model performance across devices was similar (Spectralis: accuracy = 97%, sensitivity = 94%, specificity = 95%; Cirrus: accuracy = 95%, sensitivity = 94%, specificity = 93%) (Table 3). The ROC curves for both models can be visualized in Figure 7. 

### 3.3. GA Lesion Size Classifier

The mean GA area measurements (in percentage of total scan area) when excluding GA lesions that were less than 250 um in the greatest linear dimension (including the non-horizontal linear dimensions) were significantly lower (10.85% vs. 10.95%) than the GA area measurements obtained when including GA lesions of all sizes (*p* < 0.001). When lesions smaller than 250 um were present, mean percentage difference in GA area measurement between the two groups was 11%. cRORA can be visualized in Figure 3.

## 4. Discussion

This study demonstrates the use of B-scan and *en face* ML-based approaches for automated binary detection and quantification of the presence of GA, hypertransmission defects, and pixel-level segmentation of GA area on the commonly available SD-OCT systems. AMD is the most common cause of newly acquired blindness in adults aged more than 50 years. Majority of these patients are comprised of dry AMD and GA is responsible for most of this blindness burden. Given the number of novel treatments being tested for efficacy, it is important that precise methods capable of monitoring disease progression and treatment non-invasively be developed. Current study builds on previous literature by deploying the most commonly available SD-OCT systems in clinical practice for automated GA detection and segmentation using a large and diverse dataset. In addition, the automated hypertransmission defect detection may provide a critical opportunity for detecting at-risk lesions prior to complete GA development.

The B-scan based method is a more granular approach for GA detection and segmentation as it considers the RPE loss and photoreceptor loss for the entire OCT volume on a slice-by-slice basis and is not potentially as susceptible to isolated hypertransmission defects prior to development of true cRORA. This approach is relatively novel due to lack of availability of large volumes of high-quality segmentations that are targeted to the specific outer retinal zones. There are only two other recent B-Scan based automatic GA segmentation approaches reported in literature [11]. One of these limited its training and analysis to only 5 B-scans per OCT volume [11]. The other report focused on utilizing a retinal layer independent OCT B-scan column-based approach for achieving automatic segmentation of GA with an F-score of 0.78 [12]. None of these approaches generate pixel-wise GA lesion area measurements. The B-scan model described in the current study is particularly unique as >100,000 individual B-scans with previously validated retinal multilayer segmentation were utilized in its creation and testing. Quantitative features of GA were identified using this approach as described in the CAM consensus definition for GA in AMD including RPE loss and photoreceptor loss. This yielded a high accuracy and strong correlation with ground truth for both binary detection of presence of GA and pixel-wise lesion area measurements. Further, this model performance generalized well across the two device types tested in this study with comparable performance metrics.

The *en face* methods of GA detection has been more widely explored in the literature. While the definition of GA, according to the Classification of Atrophy Meetings (CAM) consensus, is based on average B-scans, the detection of GA using *en face* OCT is a commonly deployed alternative using FAF and conventional OCT systems [13,14,15]. ML-based approaches have previously been used to segment GA lesions using *en face* images. Some studies have deployed color fundus photography as inputs for the GA lesion segmentation [16,17]. Several other studies have utilized fundus autofluorescence and *en face* SD-OCT to achieve GA lesion segmentation [18,19]. The majority of these studies use custom SD-OCT *en face* slabs or sub RPE *en face* OCT images for training the GA detection models. The native software on select OCT devices allows creation of custom slabs assessing subRPE illumination area. This has also recently been studied for GA growth monitoring [20]. While these slabs yield higher contrast for GA lesion edge and minimize artifacts from OCT [19], they can be challenging to obtain in routine clinical practice and large retrospective multi-center clinical studies. A recent study utilized sub-RPE slabs obtained on swept source (SS)-OCT to create a DL-based *en face* GA segmentation model [21]. The use of a faster swept source device allows for high-speed captures with greater depth penetration and scan density which can be useful in assessing outer retinal and choroidal pathologies [21]. However, the generalizability of such an approach to SD-OCT remains uncertain due to a relatively small patient sample obtained using a single device type that is not as widely available [21]. The current study demonstrates a high performing GA segmentation approach using standard *en face* SD-OCT images in a fully automated pipeline without requiring customized slabs. The model performance assessment yielded highly accurate lesion area measurements that correlated very well with ground truth measurements. This performance generalized well between the two devices tested, as the performance metrics were comparable (f-score of 0.83 vs. 0.81). In addition, this system provides a promising opportunity for hypertransmission defect detection and quantification. Though highly accurate for GA detection, the false positives that were observed often were hypertransmission defects in areas without complete GA/cRORA development. Future work in this area will focus on advanced segmentation based on hybrid integration of these models to distinguish between areas of hypertransmission defects without frank GA to isolate these biomarkers. 

CAM consensus definition utilizes individual B-scans to establish the 250-um size threshold for cRORA [22]. However, there has been evidence that GA lesions that appear smaller than 250 um on one B-scan may be confluent with a larger GA lesion on an adjacent B-scan [23]. This creates ambiguity in the assessment as parts of the same lesion get labelled differently (cRORA vs. iRORA). Additionally, a lesion might not have 250 um dimension in the horizontal axis but may in fact be larger in a non-horizontal dimension [19]. Therefore, lesion size assessment on *en face* OCT allows for better characterization and measurement of greatest dimension of a lesion. The current study attempts to address this challenge by automatically segmenting the greatest linear dimension of GA lesions (including non-horizontal linear dimensions) using *en face OCT-based* approach to achieve accurate and representative measurements. However, such a size assessment might not be essential in the future as the rationale of using a size threshold for cRORA, as proposed by the CAM consensus, centers on the ability to reliably identify and label lesions while minimizing grader variability [10]. Using a fully automated, machine learning based approach for GA lesion segmentation, as demonstrated in the current report, had excellent ICC values with ground truth while still measuring even small areas of GA which should be classified as cRORA, if not for the relatively conservative cutoff of 250 um established in the classification of atrophy report 3 [10]. 

The current study has several notable strengths. The large, high-quality, diverse dataset from for model training along with the use of a previously validated automated machine learning enhanced multi-layer approach for retinal layer segmentation with triple-fold quality assessment process to ensure robust ground truth generation is a major advantage of this system [7,8,9]. In addition, the current study utilizes the CAM group definitions for automatic identification of cRORA by demonstrating automatic segmentation of the greatest diameter of the GA lesion, including non-horizontal diameters. This has not been described previously in automated segmentation of GA lesions. The current study uses two different approaches for GA segmentations-B-Scan and *en face* OCT based. The described B-Scan-based approach considers the quantitative GA features (RPE loss and photoreceptor loss) from all the B-Scans in the OCT volume for each scan. Additionally, despite being tested in a diverse multi-center dataset, the *en face* approach demonstrated comparable performance to previously published reports that tested performance on much smaller datasets [15,18,19]. This also creates opportunities for hybrid approaches that can harness the strengths of both individual approaches to enhance generalizability. The potential generalizability and system agnostic aspect of this platform given its similar performance on two of the most commonly used systems for clinical trials (e.g., Spectralis, Cirrus.) for training, validation, and testing of the models allowing for robust generalizability. The model performance for both approaches demonstrated robust performance across both SD-OCT devices.

The current study is not without its limitations. The data and its analysis came from a single, quaternary-level academic institution and the results derived from this study may not generalize to other populations or clinical sites. The current study describes the use of a simpler U-Net architecture. However, the choice of architecture was made to demonstrate the utility of novel B-Scan based and *en face* SD-OCT based approaches of automated pixel-level GA segmentation.

## 5. Conclusions

The current report demonstrates two deep-learning based models capable of accurately segmenting GA lesions within B-scans and GA lesions/hypertransmission defects on *en face* OCT images, in addition to subtyping the lesions according to the CAM consensus definitions. The model performance generalized well across the different device types tested. These tools are currently being evaluated in ongoing clinical trials for additional prospective validation and direct comparison to FAF measurements, and OCT-based ground truth measurements. An exciting avenue under investigation is the potential hybrid integration of the B-scan and *en face* level systems that can allow targeted tailoring of specificity and sensitivity thresholds for screening and diagnostic applications. In addition, exploration of targeted hypertransmission defect interrogation is being actively explored.

## Figures and Tables

**Table 1:** Summary of GA detection performance for both OCT B-scan and *en face* OCT models. GA: Geographic atrophy, OCT: Optical coherence tomography.

GA Detection Performance				
Model	Accuracy	Sensitivity	Specificity	f-Score
OCT B-scan	91%	86%	94%	0.87
*en face* OCT	82%	97%	42%	0.88

**Table 2:** Summary of pixel-wise GA segmentation performance for both OCT B-scan and *en face* OCT models. ICC: interclass correlation coefficient; CI: confidence interval; GA: geographic atrophy, OCT: optical coherence tomography.

Pixel-Wise GA Segmentation Performance					
Model	Accuracy	Sensitivity	Specificity	ICC (95% CI)	f-Score
OCT B-scan	94%	90%	90%	0.88 (0.82–0.92)	0.71
*En face* OCT	96%	95%	93%	0.95 (0.87–0.99)	0.82

**Table 3:** Summary of the comparison of pixel-wise GA segmentation performance for both OCT B-scan and *en face* OCT models between the device manufacturers included in this study. Heidelberg Spectralis HRA+OCT (Heidelberg Engineering, Heidelberg, Germany) and Cirrus HD-OCT (Zeiss, Oberkochen, Germany) devices were used in this study for image capture. GA: geographic atrophy, OCT: optical coherence tomography.

Comparison of Pixel-Wise GA Segmentation Performance between Device-Manufacturers			
OCT B-Scan Model			
Model	Accuracy	Sensitivity	Specificity
Zeiss Cirrus	92%	88%	87%
Heidelberg Spectralis	97%	96%	94%
***En face* OCT model**			
**Model**	**Accuracy**	**Sensitivity**	**Specificity**
Zeiss Cirrus	95%	94%	93%
Heidelberg Spectralis	97%	94%	95%

## Data Availability

Not applicable.
